# The Apparent Lack of the Risk of Intussusception Immediately After Rotavirus Vaccination Among Japanese Infants

**DOI:** 10.3390/v16111758

**Published:** 2024-11-10

**Authors:** Wakako Kikuchi, Atsuko Noguchi, Yoko Sato, Yuuki Konno, Akira Komatsu, Satoru Tandai, Wataru Kikuchi, Shinobu Miura, Hiroshi Fukaya, Tomoaki Ohata, Hiroo Noguchi, Kenichi Matsuno, Hisayuki Tsukahara, Daiki Kondo, Masaki Komatsu, Masamichi Tamura, Hiromi Koizumi, Toyoko Nakagomi, Osamu Nakagomi, Tsutomu Takahashi

**Affiliations:** 1 Department of Pediatrics, Akita University Graduate School of Medicine, Akita-shi 010-8543, Japan; wakako1530@med.akita-u.ac.jp (W.K.); tomy@med.akita-u.ac.jp (T.T.); 2 Department of Pediatrics, Hiraka General Hospital, Yokote-shi 013-8610, Japan; ysys510@gmail.com; 3 Department of Pediatrics, Noshiro Kousei Medical Center, Noshiro-shi 016-0014, Japan; konno_y724@yahoo.co.jp; 4 Department of Pediatrics, Yokote Municipal Hospital, Yokote-shi 013-8602, Japan; agira@yokote-mhp.jp; 5 Department of Pediatrics, Odate Municipal General Hospital, Odate-shi 017-8550, Japan; tanndai@odate-hp.odate.akita.jp; 6 Department of Pediatrics, Ogachi Central Hospital, Yuzawa-shi 012-0055, Japan; wataru.k@ogachi-ikyoku.com; 7 Department of Pediatrics, Yuri-Kumiai General Hospital, Yurihonjo-shi 015-8511, Japan; smiura@fancy.ocn.ne.jp; 8 Araya Kids Clinic, Akita-shi 010-1631, Japan; 9 Department of Pediatrics, Omagari Kousei Medical Center, Daisen-shi 014-0027, Japan; fukaya0617@icloud.com; 10 Omagari Children’s Clinic, Daisen-shi 014-0022, Japan; 11 Department of Pediatrics, Fujiwara Memorial Hospital, Katagami-shi 010-0201, Japan; tomo318tibmw@sunny.ocn.ne.jp; 12 Department of Pediatrics, Kita-Akita Municipal Hospital, Kitaakita-shi 018-4221, Japan; 13 Department of Pediatrics, Oga Minato Municipal Hospital, Oga-shi 010-0511, Japan; matsu-77@poppy.ocn.ne.jp; 14 Department of Pediatrics, Kazuno Kousei Hospital, Kazuno-shi 018-5201, Japan; 15 Tsukahara Childen’s Clinic, Tsutiura-shi 300-0037, Japan; 16 Department of Pediatrics, Akita Kousei Medical Center, Akita-shi 011-0948, Japan; kondod1199@akikumihsp.com (D.K.); mclinic@kidsallergycl.jp (M.K.); 17 Higashidori Kids and Allergy Clinic, Akita-shi 010-0041, Japan; 18 Department of Pediatrics, Akita Red Cross Hospital, Akita-shi 010-1495, Japan; masamichi_tamura@akita-med.jrc.or.jp; 19 Department of Pediatrics, Akita City Hospital, Akita-shi 010-0933, Japan; nanna77nana@i.softbank.jp; 20 Akita Mental and Developmental Clinic for Children, Akita-shi 011-0946, Japan; 21 Department of Hygiene and Molecular Epidemiology, Graduate School of Biomedical Sciences, Nagasaki University, Nagasaki-shi 852-8523, Japan; tnakagom@gmail.com (T.N.); onakagom@nagasaki-u.ac.jp (O.N.)

**Keywords:** rotavirus vaccines, intussusception, risk window, incidence rate

## Abstract

Rotavirus vaccines carry a small risk of intussusception mainly 1–7 days after vaccination in the United States of America, Europe, Australia, and Latin America where the background rate of intussusception is relatively low. Such risks are undetectable in Africa and India where the background rate is the lowest. Because few studies were carried out in high-background-rate countries such as Japan, we examined how intussusception occurred in infants living in Akita prefecture, Japan, while the vaccines were sold in the private market. Between 2011 and 2018, an estimated 21,677 infants (46%) were vaccinated and 54% were not. Through a retrospective survey of medical records in 18 hospitals in the prefecture, we identified 58 infants, 28 of whom were vaccinated and 30 of whom were unvaccinated, as having intussusception that met level 1 of the Brighton criteria. Thus, the intussusception rate was 123 per 100,000 infant-years (95% confidence interval [CI]: 94–160). Despite the high rate, none developed intussusception 1–7 days after the first dose of either the monovalent human rotavirus vaccine (GSK) or the pentavalent human–bovine reassortant vaccine (MSD). The incidence rate ratio of vaccinated to unvaccinated infants between 42 and 245 days of life was estimated at 0.96 (95%CI: 0.43–2.1; *p* = 0.92). Given that over 95% of infants received the first dose before 15 weeks of age, the risk of intussusception associated with the rotavirus vaccines in high-incidence-rate countries can be reduced to a minimum by adhering to the recommended schedule at 2, 3, and 4 months of age.

## 1. Introduction

Intussusception is a pathological condition in which one portion of the intestine invaginates into an adjacent segment of the intestine, leading to a strangulating obstruction. It is a pediatric emergency in infants and children with its peak occurrence at 6–8 months of age with no apparent seasonality [1]. This classical condition captured much attention when a tetravalent rotavirus vaccine, RotaShield^®^ (Wyeth Lederle, Pearl River, NY, USA), was suspected to cause intussusception in its recipients, resulting in its withdrawal from the market in the United States of America [2]. Two succeeding rotavirus vaccines, Rotarix^®^ (GlaxoSmithKline, Rixensart, Belgium) and RotaTeq^®^ (Merck Vaccines, Whitehouse Station, NJ, USA), were shown to be safe with respect to intussusception after large clinical trials [3,4]. However, post-licensure studies showed that Rotarix had a small yet statistically significant risk of intussusception; in the first week after the first dose in Mexico [5] and in the first week after the second dose in Brazil [5]. There was also a similarly small risk of intussusception in the first week of the first dose of RotaTeq in Australia and the United States of America [6,7].

The risk of intussusception associated with the use of the second-generation rotavirus vaccines was generally perceived to be limited primarily to 1–7 days after the first dose and much less to the later doses. More specifically, one meta-analysis [8] showed that the pooled estimate of the relative risk of 1–7 days after the first dose of Rotarix and RotaTeq combined was 5.71 (95% confidence interval [CI]: 4.5–7.25), that after the second dose was 1.69 (95%CI: 1.33–2.14), and that after the third dose of RotaTeq was 1.14 (95%CI: 0.75–1.74). These conclusions were based on the results obtained from the studies conducted in middle-income countries in Latin America and high-income countries in the United States of America, Europe, and Australia [8]. The naturally occurring background incidence rates of intussusception vary across the globe and the incidence rates among infants in these countries were in a lower range: 36 per 100,000 infant-years in the Americas [9], 41 in Europe [9], and 71 in Australia [10].

More recently, however, it was reported that the risk of intussusception after administration of Rotarix was not higher than the background risk of intussusception in seven lower-income sub-Saharan African countries [11]. Soon, this observation was shown to be the case in South Africa, an upper-middle-income country in the African continent [12]. The background incidence rate of intussusception in Africa is scarce but reported to be 34 per 100,000 infant-years [9].

Another monovalent human rotavirus vaccine, containing G9P [11] Rotavac^®^ (Bharat Biotech, Hyderabad, India), showed no increased risk of intussusception within the first three weeks after any dose in India, which is also a lower-middle-income country [13]. The background incidence rate of intussusception in India is 18–20 per 100,000 infant-years [14,15]. Thus, it appears that the risk of rotavirus vaccine-associated intussusception is undetectable in countries where the background incidence rate of intussusception is very small when compared to the global mean rate of 74 per 100,000 infant-years [16].

Yet another monovalent human rotavirus vaccine containing G1P [8], Rotavin-M1^®^ (Polyvac, Hanoi, Vietnam), showed no case of intussusception within the 1–21-day risk window after the first dose and only one case on day 21 after the second dose in a pilot study conducted in Vietnam [17]. This last report from Vietnam, another lower-middle-income country, is of particular interest in that the observation was made in a country where the background incidence rate of intussusception was reported to be high [10,18,19].

The background incidence rate of intussusception among infants in Japan was previously shown to be much higher than the global average ranging from 144 to 191 per 100,000 infant-years [1,20,21,22]. Rotarix was introduced in the private market of Japan in November 2011 and RotaTeq in July 2012. Both Rotarix and RotaTeq were formally incorporated into the child immunization schedule of Japan in October 2020, and soon thereafter, the vaccine coverage became over 90%. Before the administration under the childhood immunization schedule, Rotarix and RotaTeq were sold on the private market and administered only to infants whose parents or caregivers wanted them to get vaccinated. We thought that this intermediate period was convenient to compare the occurrence of intussusception cases between vaccinated and unvaccinated infants because the numbers of vaccinated and unvaccinated infants would be comparable. So, we started a retrospective survey of intussusception by inviting all hospitals that had the capacity to provide pediatric beds in Akita prefecture. The questions we asked were twofold. Firstly, would we observe an increased number of intussusception cases occurring 1–7 days (and 1–21 days) after the Rotarix and RotaTeq vaccination? Secondly, would the incidence rate ratio of intussusception cases among vaccinated and unvaccinated infants be larger than one when the observation period was limited between 42 and 245 days of life considering the timing of the rotavirus vaccination?

## 2. Materials and Methods

A retrospective survey aimed at capturing all intussusception cases occurring among children less than 5 years of age living in Akita prefecture, Japan, was conducted by involving all 18 hospitals that had the capacity to provide pediatric beds to children living in the prefecture.

Akita prefecture has a population of 914,000 people, comprising 0.7% of the total population of Japan, and it is located on the northwestern coast of the main island. Its north, east, and south borders are separated from the neighboring prefectures by mountain ranges, and it faces the Japan Sea on its west side. Partly because of this geographic location, inter-hospital connections as well as connections between local clinics and hospitals constitute a prefecture-wide medical zone. As such, the lower likelihood of medical-care-seeking behaviors across the prefecture border allowed us to assume that patients with common childhood diseases including intussusception had been presented or treated in the medical facilities within the prefecture.

The start of the original study was from January 2001, and the observations that were made during the first 10 years (2001–2010) were previously published as information about the baseline incidence rate of intussusception in the absence of the rotavirus vaccine [1]. The design of the current retrospective cross-sectional study was the same as the earlier one except for the collection of information regarding the rotavirus vaccine history of the patients, and the study period was from January 2011 to December 2018. At the end of the study as well as two times in the middle of the study, the senior author (A.N.) asked the head pediatrician of each hospital to search the discharge database for intussusception cases by using the K56.1 code of ICD (International Classification of Diseases)-10. Then, to those who responded to her with a case(s) of intussusception, A.N. sent a standardized questionnaire about the demographic and medical information including signs, symptoms, radiologic imaging, treatment, outcome, and anything else that might be relevant such as the results of stool testing for adenovirus, rotavirus, and norovirus antigens, if completed, and asked them to fill it out by retrieving hospital charts including radiologic imaging reports. This questionnaire was developed by our research group when conducting the previous study by Noguchi et al. [1], and the same questionnaire was used in the current survey except for the questions about the rotavirus vaccines (Supplementary Files S1 and S2).

The questionnaires that were filled out and sent back from the participating hospitals were examined and adjudicated by the senior author (A.N.) in accordance with the guidelines published by the Brighton Collaboration Intussusception Working Group [23], and, as necessary, the levels of diagnostic certainty were determined by consensus of the writing committee members (W.K., A.N., T.N., and O.N.). Thus, a case of intussusception was defined as a child who was admitted to one of the 18 hospitals in Akita prefecture during the period between January 2011 and December 2018 with a diagnosis that met level one of the Brighton criteria. We included in the final analysis only those patients who were less than 1 year of age and who resided in Akita prefecture. We also made sure that the same patient was not counted more than once in the final analysis. We decided to count only the first episode of intussusception in case intussusception occurred repeatedly in a single patient (no such cases were encountered, however).

Because rotavirus vaccines were not included in the childhood immunization program during the study period, there was no official registry for rotavirus vaccines, which precluded the counting of the number of infants who received the rotavirus vaccine doses. As an alternative way of estimating a proxy of vaccinated infants, we obtained the number of doses of Rotarix and RotaTeq that were delivered from wholesale to medical institutions from the pharmaceutical companies that sold Rotarix and RotaTeq (Japan Vaccines and MSD, respectively), and the number of vaccinated infants for Rotarix and RotaTeq was estimated by assuming that all infants vaccinated with Rotarix received a full two-doses, and those with RotaTeq, a full three doses.

To calculate the incidence rate of intussusception during the study period, the total number of case patients was divided by the person-years for the study period, which was assumed equal to the sum of the number of live births from 2011 to 2018 multiplied by one (year) without adjustment for infants leaving the cohort. The Poisson model was used to calculate the 95% CIs of incidence rates. The number of live births was obtained from the vital statistics data of Akita prefecture, which was on a continuous decrease of 25% from 6658 in 2011 to 5040 in 2018 (5917 on average).

To calculate the incidence rate ratio of intussusception among the vaccinated infants and the unvaccinated infants, we counted the number of intussusception cases that occurred between day 42 (6 weeks of age) and day 245 (32 weeks and 0 days plus 21 days) of life considering the timing of the rotavirus vaccination schedule recommended in Japan and the expanded risk window (1–21 days) for intussusception that can be associated with the post-rotavirus vaccination. To calculate the denominator for the vaccinated cohort (person-time), firstly, the number of vaccinated infants without intussusception events was calculated, i.e., the estimated number of vaccinated infants minus the number of infants who developed intussusception by day 245 of life. Secondly, this number was multiplied by 203 (245 minus 42) days. Thirdly, the person-time contributed by each of the infants with intussusception was calculated by the number of days between day 42 and the onset day of intussusception. Finally, the person-time contributed by the vaccinated infants without intussusception and the person-time contributed by the vaccinated with intussusception were added to serve as the denominator for the vaccinated cohort. Similarly, to calculate the denominator for the unvaccinated cohort, we assumed that the number of live births minus the number of vaccinated multiplied by 203 days was equal to the person-time of the unvaccinated cohort. The numerators for the vaccinated and unvaccinated cohort were the number of intussusception cases between day 42 and day 245 among vaccinated and unvaccinated infants, respectively.

Statistical analysis was carried out by using STATA version 13.1 (StataCorp, College Station, TX, USA).

This study was approved by the Institutional Review Board and Ethics Committee of Akita University Graduate School of Medicine, Japan, under registration number 1339 (approved on 13 March 2017).

## 3. Results

**Figure 1:**
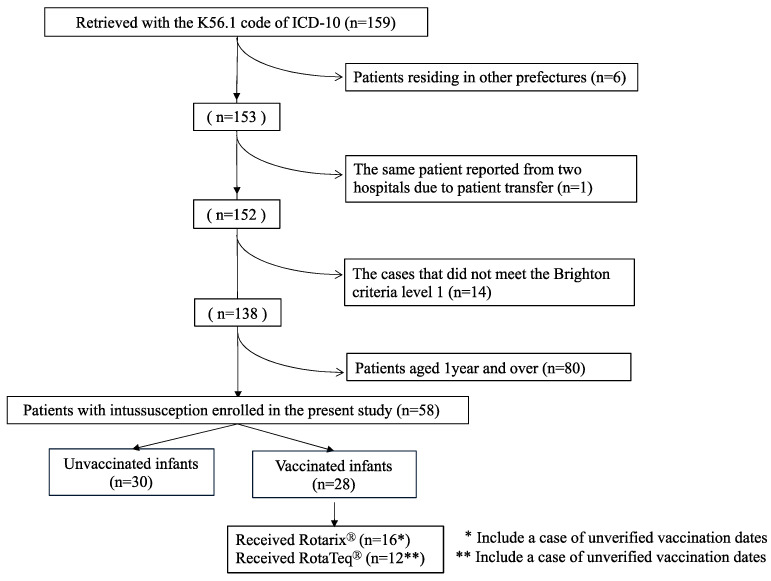
Flow chart showing the process of selection of cases subjected to final analysis.

Between January 2011 and December 2018, we retrieved 159 intussusception cases with the K56.1 code of ICD-10. There were six infants who were not living in Akita prefecture. There was one case in which the same infant was reported from two hospitals, and this duplication was corrected (Figure 1). Thus, there were 152 cases identified as having K56.1, 138 cases of which met level 1 of the Brighton criteria (Figure 1). Thus, the positive predictive value of the K56.1 code of ICD-10 for Brighton level 1 intussusception was calculated as 90.8% in this study.

Of 58 children less than one year of age (infants) with intussusception (Figure 1), 28 received either Rotarix (*n* = 16) or RotaTeq (*n* = 12). The remaining 30 infants did not receive any dose of either vaccine.

Table 1 shows the occurrence of intussusception cases over the study period, which varies from as few as 3 in 2013 to as many as 15 in 2014. Because of this variability, for which we did not have a plausible explanation, we abandoned a year-by-year analysis and, instead, used the number that summed up the eight-year study period as the denominator on which all analyses were performed. As 58 cases of intussusception occurred in the birth cohort of 47,339 when summed up for eight years, the incidence rate of intussusception among children less than one year of age living in Akita prefecture was calculated as 123 per 100,000 infant-years (95%CI: 94–160).

During the eight years from 2011 to 2018, 21,677 infants were estimated to be vaccinated with either Rotarix or RotaTeq under the assumption that full doses (two doses in the case of Rotarix and three doses in the case of RotaTeq) were given to individual recipients and that all doses delivered from the wholesale to medical institutions in Akita prefecture were administered. As the number of live births totaled 47,339 (Table 1), the average uptake rate of the rotavirus vaccine during the study period was estimated at 46%, leaving 25,662 infants unvaccinated, which accounted for 54%.

**Figure 2:**
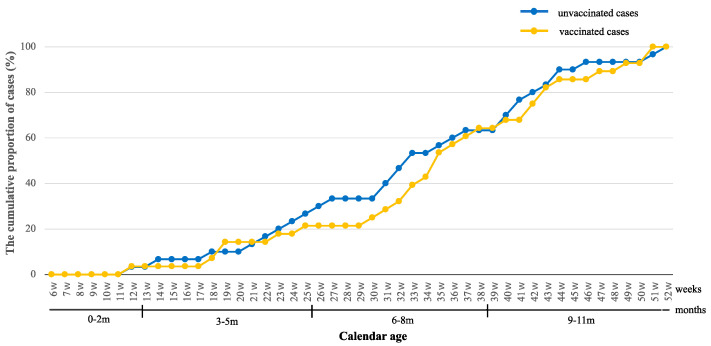
The cumulative proportion of intussusception cases among vaccinated and unvaccinated groups. The blue line and the orange line indicate the cumulative incidence expressed as a percentage of unvaccinated cases and vaccinated cases, respectively.

Figure 2 shows the cumulative incidence of intussusception cases among the vaccinated and unvaccinated groups. One notable difference between the two groups was that the accumulation of cases in the vaccinated group was slower (more flattened) than that in the unvaccinated group between 19 weeks of age and 29 weeks of age (corresponding to 5 months to 7 months of age). Consistent with this observation is the delay in the median age of onset of intussusception of about two weeks in the vaccinated group as compared to the unvaccinated group (Table 2).

While we were unable to collect the data regarding how timely the vaccine doses were given to the infants who did not develop intussusception, only 1 infant among the 28 vaccinated infants who developed intussusception received the first dose outside of the recommendation schedule; she was given the first dose at 113 days of life (16 weeks and 1 day). Thus, those who developed intussusception after either Rotarix or RotaTeq vaccination strictly adhered to the recommendation by the Japanese Society of Pediatrics (Table 3).

**Figure 3:**
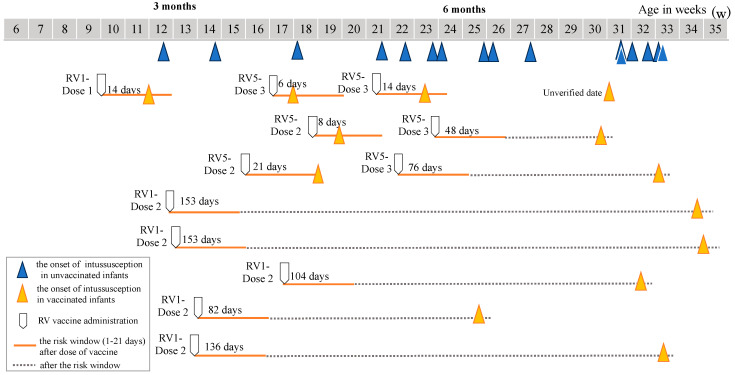
Age distribution of cases who developed intussusception during the risk window after every dose of Rotarix (indicated as RV1) and RotaTeq (indicated as RV5), respectively. Blue arrows indicate naturally occurring intussusception in unvaccinated infants; orange arrows indicate intussusception in vaccinated infants; orange lines indicate the risk window (1–21 days) after a dose of the vaccine; and gray dotted lines indicate the duration after the risk window.

Next, we examined at what time intussusception occurred in the vaccinated infants with respect to the risk windows after the vaccine doses and the age band to which they belonged at the onset of intussusception. There were 26 vaccinated infants with verified vaccination records, and 21 developed intussusceptions outside the risk window. Thus, there were five infants who developed intussusception within the risk window, but it is notable that none occurred 1–7 days after the first dose of either Rotarix or RotaTeq. The only case that occurred after the first dose was a boy who developed intussusception 14 days after receiving Rotarix when he was in the 0–2-months-of-age band (Figure 3). On the other hand, the only case that occurred within the 1–7-day risk window was a boy who developed intussusception 6 days after the third dose of RotaTeq when he was in the 3–5-months-of-age band (Figure 3). There were three more cases of intussusception occurring in the 8–21-day risk window, all of whom received either the second or the third dose of RotaTeq when they were in the 3–5-months-of-age band (Figure 3).

Next, we compared the incidence rate of intussusception among the vaccinated with that of the unvaccinated irrespective of whether the intussusception occurred within the risk window. To do this, we limited the analysis to infants who were between 42 days (6 weeks) and 245 days (32 weeks plus 21 days) of life, taking into consideration the timing of rotavirus vaccination allowed according to the infant immunization schedule in Japan. The incidence rate ratio of intussusception was estimated at 0.96 (95%CI: 0.43–2.1; *p* = 0.92) (Table 4).

## 4. Discussion

In this eight-year study, an estimated 21,677 infants living in Akita prefecture were vaccinated, of whom, 28 infants developed intussusception. However, none occurred 1–7 days after the first dose, and only one case occurred 6 days after the third dose of RotaTeq. Even when the risk window was expanded to 1–21 days after vaccination, only one case occurred 14 days after the first dose of Rotarix and three cases occurred 8–21 days after the second and third doses of RotaTeq (Figure 3).

Our observation that there was no intussusception case 1–7 days after the first dose among either vaccine recipients is not consistent with the widely accepted perception that an increased risk of intussusception exists immediately after the administration of Rotarix and RotaTeq, although the sample size in our study was small to draw a definitive conclusion.

Interestingly, this apparent lack of an increased risk of intussusception is in good agreement with the studies conducted in Africa, India, and some Asian countries in that there was no excess risk of intussusception observed after the administration of Rotarix, Rotavac, or Rotavin-M1. These vaccines are different from each other, but all are monovalent live-attenuated rotavirus vaccines deriving from human rotavirus strains. In the post-licensure studies, two oral doses of Rotarix and Rotavin-M1 were given at 6 and 10 weeks of age in African countries [11,12], and at 2 and 3 months of age in Vietnam [17], whereas three oral doses of Rotavac were given at 8, 13, and 18 weeks of age in India [13]. In contrast, two doses of Rotarix were administered at 2 and 4 months of age, and three doses of RotaTeq were administered at 2, 4, and 6 months of age in the United States of America [7].

The countries where virtually no increased risk of intussusception was previously observed in association with the administration of rotavirus vaccines are limited to middle-income countries, and some plausible explanatory hypotheses were proposed [11,12,13]: lower immunogenicity, and hence lower efficacy of oral vaccines than in high-income countries due to differences in gut microbiomes, co-administration of oral polio vaccines reducing the multiplication of rotavirus vaccine viruses in the gut, mothers’ milk containing a higher concentration of anti-rotavirus antibodies, the earlier age at which to administer the doses of rotavirus vaccine compared to high-income countries, and so on. Of these, the earlier age of vaccine administration is a common factor in the situation in Japan where Rotarix was administered at 2 and 3 months of age and RotaTeq at 2, 3, and 4 months of age.

Taken together, the apparent lack of an increased risk of intussusception appeared to result from the practice that the first dose of rotavirus vaccines was given to >95% of infants between 6 weeks and 14 weeks and 6 days, the age band when the naturally occurring intussusception rate is the smallest. Although we did not have data about the percentage of the vaccinated infants without intussusception receiving the first dose according to the immunization schedule, our previous study conducted in a part of Akita prefecture between 2013 and 2015 showed >95% of infants were vaccinated adhering to the immunization schedule [24]. Our observation is also in good agreement with a recent study conducted in Hokkaido prefecture, Japan, in which none of the intussusception cases occurred within the one-month period after any dose of the rotavirus vaccine, although the paper did not disclose the number of intussusception cases occurring in infants aged less than 6 months [25].

At a glance, the distribution of intussusception cases, particularly in the risk window in the vaccinated group, seems different between our study (only five within the 1–21-day risk window) and the post-marketing monitoring study of intussusception carried out in Japan by GSK Vaccines [26]. In the GSK study, clustering of intussusception cases 1–7 days after both the first and the second dose of Rotarix was evident from which they calculated an observed/expected ratio (relative risk) of 2.96 (95%CI: 1.42–5.45) after the first dose, whereas such clustering was lacking in our study. This difference was most likely caused by the difference in the size of the denominator from which cases of intussusception arose (and were reported). The source of the GSK study was the company’s database on the spontaneous adverse event report, and approximately 601,000 doses of Rotarix were shipped during their study period, corresponding to approximately 300,500 infants under the same assumption that those who were vaccinated received two doses [26]. Specifically, they described that there were 27 intussusception cases that met levels 1–3 of the Brighton criteria. There must be many intussusception cases that were not reported because the cases were judged unrelated to the vaccine administration due to the longer interval between the onset of intussusception and vaccination (reporting bias). On the other hand, our sample size was small. More importantly, however, we captured all cases of intussusception that occurred in the entire prefecture and collected intussusception cases independent of the use of rotavirus vaccines.

The difficulty in distinguishing naturally occurring intussusception cases from those that are triggered by the administration of vaccine doses is illustrated in Figure 3. All cases of intussusception that occurred within the 1–21-day risk window (indicated by orange arrows in Figure 3) were distributed within the 3–6 months-of-age band where a similar number of naturally occurring intussusception among unvaccinated infants was observed (indicated by blue arrows in Figure 3). When the 46% uptake rate of the rotavirus vaccine is taken into consideration, the denominators of vaccinated and unvaccinated infants were similar in size; thus, it is instinctively clear that at least a huge excess of vaccine-associated intussusception did not occur in this population. To further address this hypothesis, we calculated the ratio of the intussusception rate among the vaccinated infants to that among the unvaccinated infants between day 42 and day 245. The incidence rate ratio was 0.96 (95%CI: 0.43–2.1; *p* = 0.92), which suggests a lack of an increased risk associated with rotavirus vaccination.

This study has several limitations. First, the sample size was small. The small sample size was partly because of the rapidity at which the number of children born in Akita prefecture was declining. According to the most recent vital statistics, the number of live births in Akita prefecture in 2023 was 3611 despite the fact that it is the sixth largest prefecture in terms of area in Japan.

Second, we were not able to obtain an accurate number of infants who were vaccinated with Rotarix and RotaTeq and had to rely on the number of vaccine doses to be delivered to medical institutions in Akita prefecture from the wholesale; we also made a further assumption that those who were vaccinated were given full doses. However, we believe that the number of doses delivered to the medical institutions was almost perfectly administered to eligible infants because medical institutions tend to not keep stocks in their own facilities as returning unused vaccines to the wholesale or pharmaceutical companies is forbidden.

Third, as a result of not collecting demographic and other information, it remained an assumption that the denominator from which cases of intussusception occurred was similar in various demographic, social, and other characteristics to those who did not develop intussusception during the observation period.

Fourth, what we disregard from person-years in calculating the denominator were 940 and 78 infants (an annual average of 118 and 17 infants) who emigrated and died, respectively, during their first year of life, and left the birth cohort. As the denominator was 47,339, the contribution of the fraction leaving the birth cohort was eventually as small as 1% or so.

## 5. Conclusions

A retrospective survey of intussusception in the entire Akita Prefecture, Japan, over eight years during which 46% of the infants were vaccinated with either Rotarix or RotaTeq found no increased risk of intussusception immediately after vaccination. Strict adherence to the vaccination schedule is highly recommended to dispel the concern of intussusception risks associated with rotavirus vaccines even in countries where the background rate of intussusception is high.

## Figures and Tables

**Table 1:** Number of cases of intussusception among vaccinated and unvaccinated infants (age less than one year) and the number of live births, 2011–2018.

Year	Vaccinated (Cases)	Unvaccinated (Cases)	Number of Live Births
2011	0	7	6658
2012	0	5	6543
2013	1	2	6177
2014	5	10	5998
2015	4	3	5861
2016	12	1	5666
2017	2	2	5396
2018	4	0	5040
2011–2018	28	30	47,339

**Table 2:** Ages of vaccinated and unvaccinated infants at the onset of intussusception.

	Number of Infants	Median (Weeks)	Interquartile Range
Vaccinated	28	35.6	31.0–42.8
Unvaccinated	30	33.2	26.0–41.8

**Table 3:** The recommended schedule of rotavirus vaccination, the number of cases of infants adhering to the restricted range of the first dose, and the weeks in age at each dose of the vaccines.

	Rotarix^®^		RotaTeq^®^		
	Dose 1	Dose 2	Dose 1	Dose 2	Dose 3
Vaccination schedule recommended in Japan	6w-14w6d	(27 days after dose 1)-24w0d	6w-14w6d	(27 days after dose 1)-28w0d	(27 days after dose 2)-32w0d
Number of patients vaccinated on schedule at dose 1	14/15 *		11/11		
Median age in weeks at vaccination	9.9	14.4	10.7	16.0	20.3
Interquartile range	9.4–12.1	13.6–17.3	9.1–12.2	13.5–17.8	17.9–22.3

* One patient received dose 1 at the age of 16 weeks and 1 day.

**Table 4:** The occurrence of intussusception among vaccinated and unvaccinated infants between 42 days and 245 days of life.

	Vaccinated Infants	Unvaccinated Infants
Number of intussusception cases	13	16
Estimated number of infants	21,677	25,646
Person-days	4,399,698	5,208,365
Incidence rate (per 100,000 person-years)	108	112
Incidence rate ratio	0.96 (95% confidence interval: 0.43–2.1)	

## Data Availability

The raw data supporting the conclusions of this article will be made available by the authors upon request.

## Supplementary Materials

The following supporting information can be downloaded at https://www.mdpi.com/article/10.3390/v16111758/s1, Supplementary File S1: Questionnaire for cases of intussusception, page 1. Supplementary File S2: Questionnaire for cases of intussusception, page 2.
