# Supplementary material for: The Apparent Lack of the Risk of Intussusception Immediately After Rotavirus Vaccination Among Japanese Infants
Source: Viruses. 2024 Nov 10;16(11):1758. doi: 10.3390/v16111758 (PMC11599134; doi:10.3390/v16111758)
Supplement: Supplementary file 1 [file viruses-16-01758-s001.zip › Supplementary file 1.pdf]

Questionnaire on cases of intussusception

Affiliation	
Dr's Name	

Anonymized number	
Sex	male ・ female
Age	yy/mm
Adress(city only)	

Date of birth	yy/mm/dd
Date of onset	yy/mm/dd
Date of hospital visit	yy/mm/dd
Date of diagnosis	yy/mm/dd

＜Evidence for diagnosis：Level1＞（The Bribhton Collaboration case definition Vaccine 22:569-574,2004）

１）Abdomial ultrasound

Ultrasound findings of intussusception（target sign、pseudokidney sign、sandwitch sign ）	(Yes / No / Unknown)
Ultrasound findings after non-invasive reduction	(Disappearance of intussusception / Remaining intussusception / other)

２）Enema

Method(contrast agent）	（ Barium ・ Gastrografin ・ Air ・ not performed ）
The demonstration of intussuscepti	(Yes / No / Unknown)
If yes, types of findings	（ Crab claw sign ・ other : ）
Outcome	（ Reduction / Impossible reduction / Other： ）

3）Surgery （ Performed / Not performed / Unknown ）

The demonstration of intussuscepti	(Yes / No / Unknown)
If yes, that prosedure	（Reduction by the Hatchinson technique / intestinal resection: area cm)

＜Evidence for diagnosis：: Level 2-3＞

Vomiting	(Yes （→ bilous ・ not bilous ） / No / Unknown)
Abdominal distension	(Yes / No / Unknown)
Abnormal or absent bowel sounds	(Yes / No / Unknown)
Bloody stool	(Yes / No / Unknown)
Abdominal pain	(Yes / No / Unknown)
Abdominal mass	(Yes / No / Unknown)
Dance sign (intestinal prolapse)	(Yes / No / Unknown)
Abdminal CT	（ Performed / Not performed / Unknown ）
CT findings	（ ）
