# Supplementary material for: The Apparent Lack of the Risk of Intussusception Immediately After Rotavirus Vaccination Among Japanese Infants
Source: Viruses. 2024 Nov 10;16(11):1758. doi: 10.3390/v16111758 (PMC11599134; doi:10.3390/v16111758)
Supplement: Supplementary file 1 [file viruses-16-01758-s001.zip › Supplementary file 2.pdf]

Questionnaire on cases of intussusception

＜Accompanying symptoms＞

Diarrhea (Yes / No / Unknown)

If yes, date of onset (yy/mm/dd) (or before or after the intussusception)

Pyrexia (37.5℃ or more) (Yes / No / Unknown)

Upper respiratory infection (cough rhinorrhea, throat pain ,) (Yes / No / Unknown)

Others ( )

<Rotavirus vaccination＞

Type of vaccine (Unvaccinated ・ Rotarix (RV1) ・ Rotateq(RV5))

Number of vaccitations (None ・ Once ・ Twice ・ Three times)

Date of vaccine (1st yy/mm/dd, 2nd yy/mm/dd, 3rd yy/mm/dd)

＜Screening of infection＞

Fecal culture (Performed / Not performed / Unknown)

If performed, results (Pathogen 「 」 ・ not detected)

Rapid screening of virus in stool (Rotavirus ・ Adenovirus ・ Norovirus ・ Other ・ Not tested)

Result with positive (Rotavirus ・ Adenovirus ・ Norovirus ・ Other ・ All negative)

Serological examination (antibody titer, etc.) (Performed / Not performed / Unknown)

If performed, results ( )

＜Gastrointestinal malformations＞ (Yes / No / Unknown)

If yes, disease is（Meckel's diverticulum, Intestinal duplication, Polyps, Tumors, Other( ) ）

＜Recurrence of intussusception＞ (1st time / 2nd time / 3rd time / other :)

＜Outcome＞ (Cure ・ Transfer to the other hospital for reduction ・ Transfer to the other hospital for surgery ・ Other)

＜Days of hospitalization＞ (days)
